# Patellar Fractures: A Clinical Narrative Review

**DOI:** 10.7759/cureus.94600

**Published:** 2025-10-14

**Authors:** Zeeshan M Ali-Qazalbash, Ahmad A Quzli, Zainab Ali-Qazalbash, Sondos A Alkhatib, Rawand A Quzali

**Affiliations:** 1 Trauma and Orthopaedics, Wirral University Teaching Hospital National Health Service (NHS) Foundation Trust, Birkenhead, GBR; 2 Emergency Medicine, Fauji Foundation Hospital, Rawalpindi, PAK; 3 Internal Medicine, Liverpool University Hospitals National Health Service (NHS) Foundation Trust, Liverpool, GBR; 4 Physiotherapy, Abu Dhabi Health Services Company (SEHA), Abu Dhabi, ARE

**Keywords:** cannulated screw, knee biomechanics, knee extensor mechanism, patella fracture, patella plate, tension band wire, the knee joint

## Abstract

Patellar fractures are common knee injuries that can severely compromise the extensor mechanism of the knee. They may result from direct trauma, indirect quadriceps contraction, or high-energy impacts. A sound understanding of their anatomy, biomechanics, and fracture patterns is essential for accurate diagnosis and effective management.

This review summarises the current evidence on patellar fractures, covering anatomy, clinical evaluation, imaging, classification, and treatment. Both conservative and surgical treatments are discussed, such as tension-band wiring, screw fixation, plate osteosynthesis, and patellectomy, along with rehabilitation protocols and outcomes.

Fractures with minimal displacement and an intact extensor mechanism can generally be treated successfully without surgery, with immobilisation and early physiotherapy, and many patients regain good function. Displaced or complex fractures generally require operative fixation, and outcomes have been enhanced by advances in imaging and plating technology. The most frequent complications are hardware irritation and re-operation; quadriceps weakness and post-traumatic arthritis can also affect long-term function.

Optimal management of patellar fractures depends on careful consideration of fracture pattern, extensor mechanism integrity, and patient factors. Individualised treatment and careful follow-up allow most patients to achieve satisfactory functional recovery, although potential complications should be expected and managed.

## Introduction and background

Patellar fractures are relatively common knee injuries, accounting for a substantial proportion of fractures around the knee. In a large epidemiological analysis of adult fracture patterns, patellar fractures accounted for nearly one-third of all knee fractures [1]. Although they occur with an overall incidence of about 1% of all skeletal injuries, their clinical significance is considerable due to the patella’s central role in the extensor mechanism of the knee joint [2]. The patella is the largest sesamoid bone in the human body, embedded within the quadriceps tendon and articulating with the femoral trochlea. Its primary biomechanical function is to act as a hypomochlion, a mechanical fulcrum that increases quadriceps leverage and enhances knee extension efficiency [2]. Because of its subcutaneous position and the high mechanical loads transmitted across the knee joint, the patella is particularly prone to injury from direct trauma, indirect tensile forces, or combined mechanisms [2]. The demographic distribution of patellar fractures is typically bimodal. They are frequently seen in older women following low-energy falls, often in the context of osteoporosis, but may also result from high-energy trauma in younger patients, where they are commonly associated with concomitant injuries [1]. These epidemiological patterns reflect part of a broader shift in the incidence of knee fractures, with increasing rates of low-energy injury in an ageing population [1]. Despite their frequency, patellar fractures present ongoing challenges in classification and management. Key challenges include interobserver variability in classification, particularly with comminuted and distal pole patterns, and a lack of consensus on fixation strategies across fracture types, leading to heterogeneous treatment pathways and outcome reporting [2]. Conventional radiographs remain the mainstay of diagnosis, yet computed tomography (CT) is increasingly recognised for its role in detecting complex fracture patterns. Treatment options range from conservative non-operative approaches in stable fractures with preserved extensor mechanism to a variety of surgical strategies aimed at restoring the extensor mechanism and preserving the patellofemoral joint surface [2]. The purpose of this narrative review is to provide a comprehensive overview of patellar fractures, including applied anatomy, biomechanics, diagnosis and radiological assessment, classification systems, management options, surgical strategies, postoperative care, outcomes, and complications.

## Review

Applied anatomy

The patella is the largest sesamoid bone in the body, located anterior to the knee joint within the patellofemoral groove. It is attached to the quadriceps muscle superiorly by the quadriceps tendon and inferiorly to the patellar tendon. The patellar retinaculum also attaches to it and is formed by fibres from the fascia lata, vastus medialis, and vastus lateralis. In addition, the patella has ligamentous attachments, the medial patellofemoral ligament (MPFL), primarily providing static stability to prevent lateral translation of the patella. The posterior surface of the patella is articular and is covered by the thickest cartilage in the body (up to 1 cm in thickness). This articular surface is divided into a smaller medial and a larger lateral facet, separated by a vertical ridge. Because of its subcutaneous position and high levels of mechanical strain exerted by the extensor mechanism, the patella is prone to injury and displacement [2].

Aetiology and biomechanics

A diverse set of mechanisms can result in patellar fractures including direct trauma usually in the form of direct anterior force, indirect transmission of forces leading to avulsion fractures, high-energy trauma, or a combination of mechanisms - all of which have variable effects ranging from partial or complete loss of the extensor mechanism and can be associated with concomitant injuries, including open fractures [3]. Periprosthetic patellar fractures may also occur and remain a significant cause of postoperative complications following total knee arthroplasty [4,5]. Multiple studies have shown low-energy trauma to the patella directly to be the most common cause of patellar fractures [5], with these fractures being shown to be most common in older women (over 60 years old). Some consider patellar fractures in this population to be related to osteoporosis and hence can be considered as fragility fractures [6-8]. The distribution is bimodal, with these injuries occurring either as fragility fractures in older osteoporotic women, or as a result of high-energy trauma in younger patients, often present with associated injuries. The patella is subjected to complex biomechanical forces through the normal range of motion of the knee. During extension, the pull of the quadriceps tendon generates tensile forces on the patella. Conversely, during flexion of the knee, compressive forces act on the patella through the quadriceps and patellar tendons, along with three-point bending forces, which may result in the patella failing and fracturing in compression in cases of strong sudden knee flexion during contraction of the quadriceps [9]. Tensile forces from the extensor mechanism can cause indirect avulsion or transverse fractures, which may be complicated by displacement [9]. Direct anterior blunt force may cause transverse patellar fractures that disrupt the extensor mechanism, typically following a fall or high-energy trauma such as a dashboard injury in a motor vehicle collision.

Diagnosis

Diagnosis of these injuries is based on a detailed analysis of the mechanism of injury, physical examination findings, as well as radiological investigations. If high-energy trauma is suspected, management should follow advanced trauma life support (ATLS) principles [10]. If an open fracture is suspected or confirmed, initial management should follow British Orthopaedic Association Standards for Trauma (BOASt) guidelines, the wound should be photographed, covered in saline soaked gauze, immobilised in a brace, and appropriate medication administered including intravenous antibiotics as per local Trust guidance and tetanus prophylaxis if needed. In such cases, early discussion with orthoplastic surgeons should be considered to ensure adequate soft-tissue coverage if required [11]. A thorough physical examination should be performed, looking for features suggestive of patellar fractures, such as bruising, swelling, knee deformity, a palpable gap in the patella, effusion indicating haemarthrosis, or loss of the extensor mechanism (evident as an inability to perform a straight-leg raise) [5]. An intact extensor mechanism does not exclude a patellar fracture, as an intact parapatellar retinaculum may permit extension despite complete or multifragmentary patellar fractures [3]. Possible associated injuries should be considered based on the mechanism of injury, such as open fractures (particularly with high-energy trauma), long bone injuries, and pelvic or acetabular fractures associated with dashboard injuries. A complete neurovascular examination of the affected limb should be performed.

Radiological assessment

Radiological investigations are essential to confirm the diagnosis. Plain radiographs are generally sufficient, with two views (anteroposterior and lateral) usually adequate. Lateral radiographs are particularly valuable for assessing fragment displacement, aiding classification and guiding management decisions. In some cases, skyline and oblique views may help identify additional fracture lines and small fragments. If no clear fracture line is seen but clinical or radiological evidence of haemarthrosis or lipohaemarthrosis is present, an occult bony injury should be suspected and further imaging performed [5]. An important differential diagnosis on assessing radiographs for patellar fractures is a bipartite patella. This arises from failure of fusion of the primary and secondary ossification centres of the patella, most often in the superolateral portion. It can be distinguished from acute fractures by its well-corticated, sclerotic edges, and is bilateral in about 50% of cases [12]. Plain radiographs may underestimate fracture complexity. Computed tomography (CT) is increasingly used to assess these injuries, with studies showing that treatment plans change in up to 50% of cases based on CT findings. For example, distal pole fractures of the patella are often underappreciated on conventional imaging [13].

Classification

**Figure 1:**
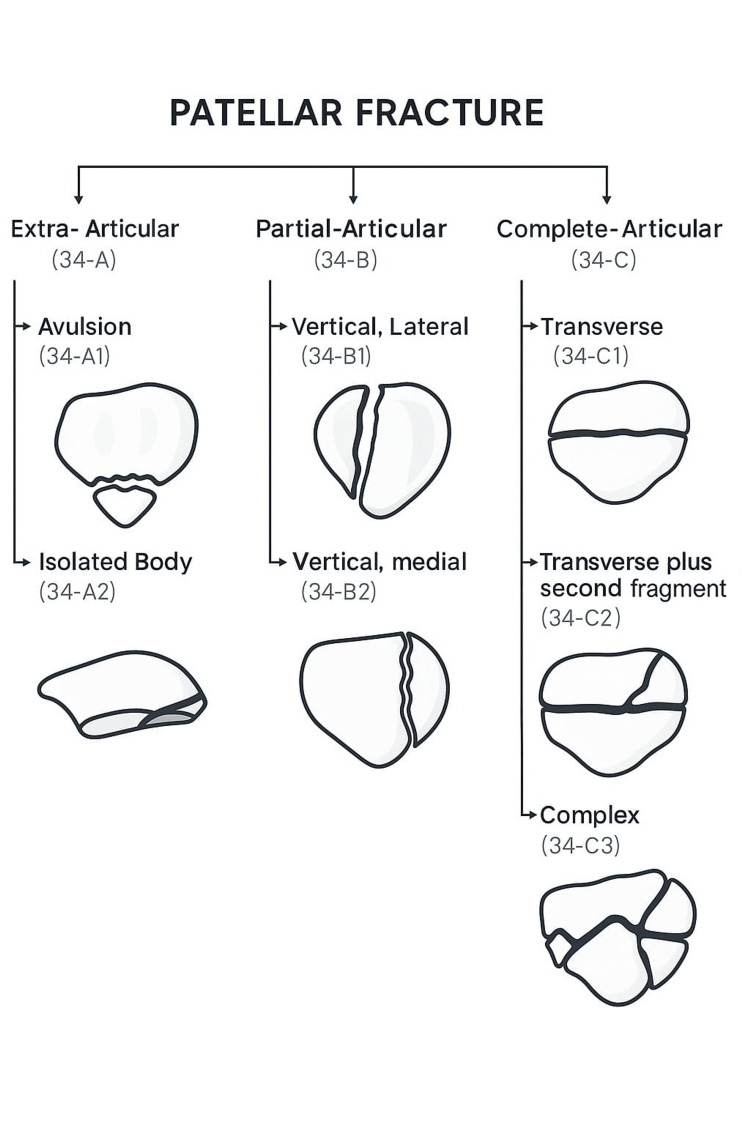
Classification of patellar fractures as per AO/OTA. AO/OTA classification of patellar fractures [14]. Illustration created by Dr. Ahmad A. Quzli based on the AO/ASIF classification system. AO/OTA: Arbeitsgemeinschaft für Osteosynthesefragen/Orthopaedic Trauma Association; AO/ASIF: Arbeitsgemeinschaft für Osteosynthesefragen/Association for the Study of Internal Fixation.

Multiple classification systems exist for patellar fractures. Historically, these fractures were classified using plain radiographs, which delineated fracture fragments and allowed classification by location, pattern, comminution, and displacement [5]. Although descriptive of the likely mechanism of injury, this system did not guide treatment decisions. The Arbeitsgemeinschaft für Osteosynthesefragen/Orthopaedic Trauma Association (AO/OTA) classification system is now more widely used. It categorises fractures as extra-articular (34A), partial articular (34B), or complete articular (34C), with further subclassification based on comminution, as shown in Figure 1 [14]. In practice, the AO/OTA categories help frame initial management. The 34A (extra-articular) patterns with preserved extensor function and minimal displacement are often suitable for non-operative care. The 34B (partial articular) patterns usually require fixation to restore joint congruity. The 34C (complete articular or comminuted) patterns are the most challenging and typically require operative fixation tailored to fragment morphology and bone quality, and in rare cases may necessitate partial patellectomy when stable reconstruction is not feasible [14,15].

Management

Management of patellar fractures depends on several factors, including thorough physical and neurovascular examination, the presence of concomitant injuries, and whether the fracture is open or closed. Initial management includes initial assessment and plain radiographs. Acutely, the knee is usually immobilised in extension using a cricket pad splint, a hinged knee brace locked in extension, or a cast while awaiting confirmation of diagnosis. Immobilisation in extension prevents ongoing quadriceps and patellar tendon forces from further displacing fracture fragments. In general, these fractures can be managed either non-operatively or operatively depending on multiple factors and indications as shown in Table 1 [15]. Goals include restoration of the extensor mechanism primarily, and prevention of patellofemoral joint post-traumatic osteoarthritis secondarily. Over time, there has been an increase in operative management of these fractures, with operative figures increasing to 75.78% of patellar fractures from 53.16% over the 14-year period of a large study in Germany [16]. One possible reason for this trend is the increasing utilisation of computed tomography (CT) for patellar fractures, which has improved characterisation of fracture patterns and identification of complexity. In a prospective level I trauma centre study of 41 patellar fractures, four fellowship-trained trauma surgeons classified fractures and planned treatment from radiographs and then repeated the process with CT; adding CT upgraded the AO/OTA classification in 27 patients (66%) and altered the planned surgical strategy in 20 patients (49%), with a distinctive comminuted distal pole pattern frequently under-recognised on radiographs [13].

**Table 1:** Indications for operative and non-operative management of patellar fractures Source: Steinmetz et al. [15].

Management	Indications
Non-operative	Intact extensor mechanism
	Minimally displaced transverse or vertical fracture pattern
	Significant medical comorbidities
Operative	Open fracture
	Displaced fracture (>4 mm fracture gap or >2-3 mm step)
	Disrupted extensor mechanism
	Presence of loose bodies
	Comminuted / complex fracture pattern

Non-operative management

Patellar fractures with intact extensor mechanism or minimal displacement can be managed non-operatively in splints. Minimally displaced fractures are defined as having a fracture gap of <3 mm and <2-3 mm of cortical step-off [15]. In such stable patterns, early, supervised mobilisation is associated with favourable return to function and low nonunion in observational cohorts [5,15]. In addition, this option can be considered for displaced fractures in comorbid or frail patients and can be trialled initially, with follow-up radiographs at one week to assess displacement. Early weight-bearing in extension and supervised physiotherapy can be instituted in these stable patterns, typically using a brace locked in extension with early range of motion and an early radiographic check in the first one to two weeks to detect any secondary displacement [5,14,15]. Initially, the leg is kept in full extension for the initial one to two weeks, followed by gradual incremental increases in range of motion, and resistance exercises allowed from six weeks on [15].

Surgical strategies

Various surgical options exist for the management of patellar fractures, including open reduction and internal fixation with various constructs - such as tension-band constructs, cannulated screws, plate fixation or patellectomy - which can be partial or complete. Patella preservation is generally preferred when feasible, given its role in extensor mechanics and functional outcomes, and patellectomy is reserved for non-reconstructable cases [5]. Consensus is yet to be reached regarding the optimal surgical option for fixation, however more complex fracture patterns are more commonly being managed with plate osteosynthesis.

Open reduction internal fixation (ORIF), as the name entails, involves initially exposing the fracture site and performing a temporary reduction using clamps and K-wires. Once the fracture is adequately reduced, fixation can be undertaken using the desired method and construct.

**Figure 2:**
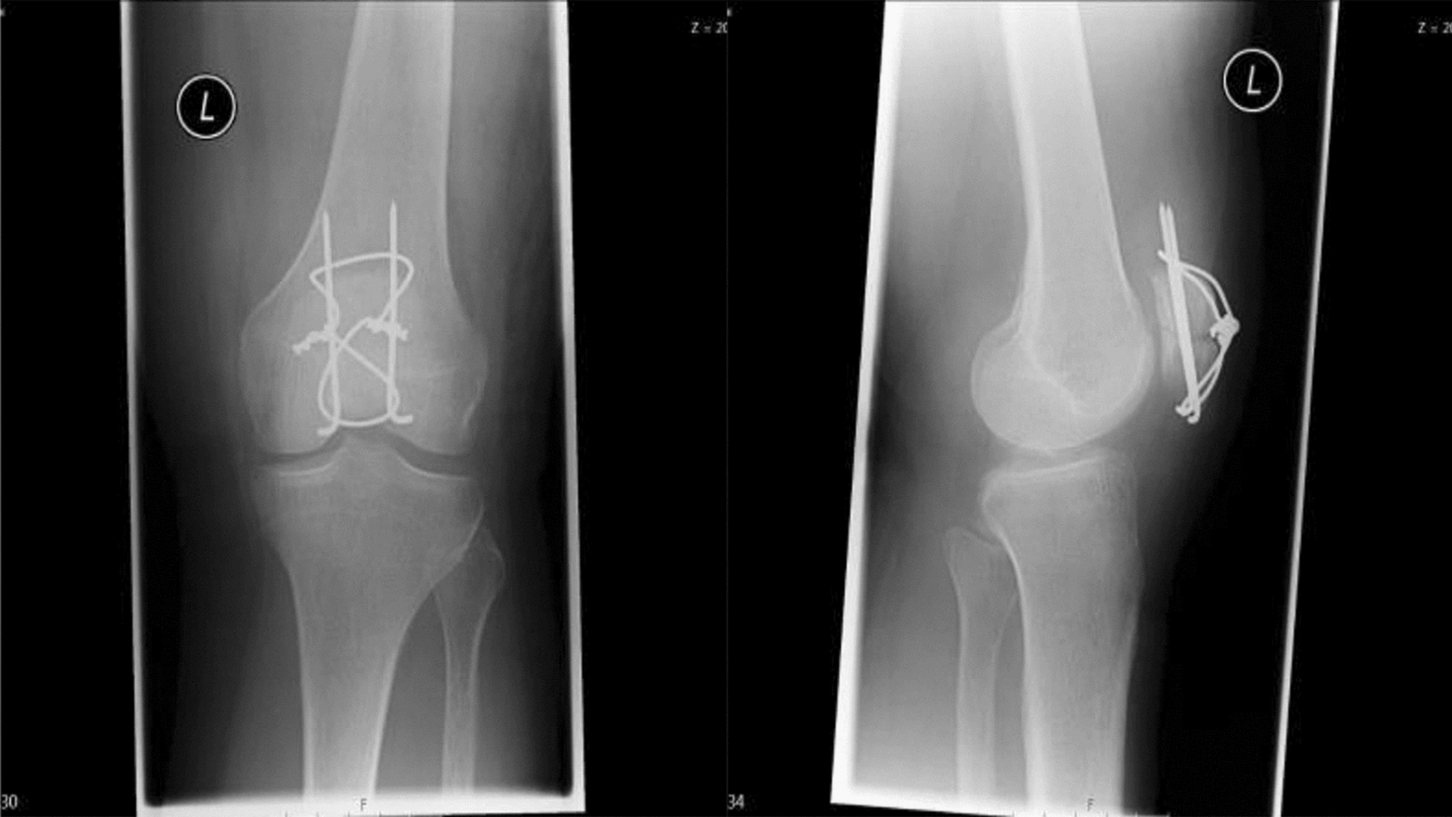
Anteroposterior and lateral radiographs of a transverse patellar fracture following operative fixation with tension-band wiring. Reproduced from Pesch et al. [28], licensed under Creative Commons Attribution 4.0 International License (CC BY 4.0).

Tension-band constructs are the most commonly employed method for fixation of these fractures (see Figure 2). These constructs are made with two Kirschner wires or cannulated screws reducing the fracture, connected in a figure-of-eight fashion via wire or suture in such a way that the tensile force generated by the quadriceps and patellar tendons is converted into a compressive force at the articular surface, allowing healing of the fracture. If wires are used, they are then cut and buried under the skin to avoid irritation. This is known as the modified anterior tension band (MATB) technique. Using cannulated screws instead of K-wires increases the overall strength of the construct and is biomechanically superior [17,18]. The main drawback of tension-band wire (TBW) constructs is the high rate of re-operation due to hardware irritation caused by the metalwork, often requiring re-operation for their removal [19].

**Figure 3:**
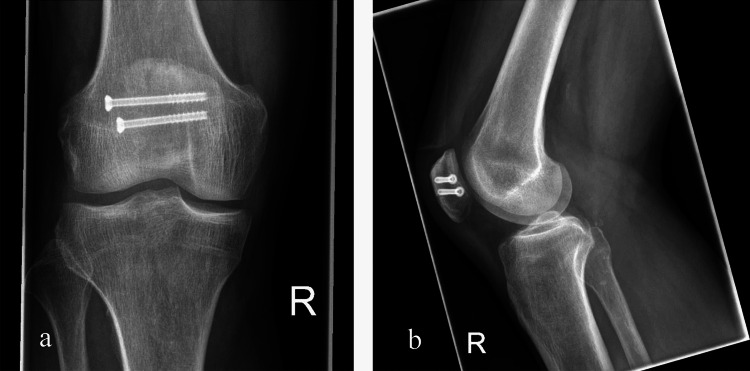
Anteroposterior (a) and lateral (b) X-ray views of a simple transverse patellar fracture managed operatively using screw fixation Reproduced from Pesch et al. [28], licensed under Creative Commons Attribution 4.0 International License (CC BY 4.0).

Fixation with cannulated screws is another option and can be done using a small incision (see Figure 3). This follows the principles of compression to allow healing; however, the risk of migration of fragments remains due to the cancellous bone of the patella. In addition, studies have shown that the biomechanics of such a fixation may be weaker than a tension band construct, with more displacement of the fracture at certain loads and failure at lower loads compared to TBW fixation [18].

**Figure 4:**
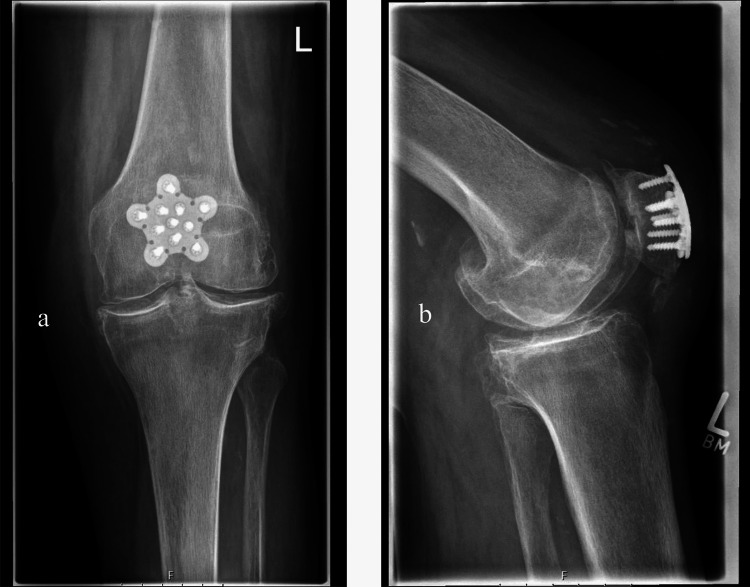
Anteroposterior (a) and lateral (b) X-rays showing a multifragmentary patellar fracture following operative fixation with a locking plate Reproduced from Pesch et al. [28], licensed under Creative Commons Attribution 4.0 International License (CC BY 4.0).

Plate fixation is becoming increasingly popular, with a large study in Germany highlighting a significant increase in usage of plate rather than tension band wiring constructs, particularly for complex fracture patterns, due to better biomechanics and the ability to fix multiple fragments [16]. Most reported advantages are supported by biomechanical studies [20-22]. Clinical evidence from recent systematic reviews and cohort work suggests that locking plate constructs can achieve high union rates, fewer implant related complications or removals, and satisfactory functional scores compared with traditional tension band techniques, particularly in comminuted 34C patterns [23-25]. Direct randomised head-to-head evidence comparing locking plates with tension band techniques is not yet available, and no meta-analysis of randomised trials has been published to date. Several forms of locking plate are available, which reduce postoperative displacement and demonstrate superior biomechanics compared with both wire and screw-suture tension-band constructs [20-22]. Reported complications include hardware prominence or soft-tissue irritation that may require removal [26]. In addition, these plates provide more versatility, allowing placement of multiplanar screws and can be useful in osteoporotic bone [16] (see Figure 4).

If preservation of the patella is not feasible, patellectomy may be considered. Patellectomy refers to the partial or complete resection of the patella and is indicated in certain circumstances such as severely comminuted fractures not amenable to fixation, or malignancy. Patellectomy reduces the effective extensor lever arm and increases quadriceps demand, which has been reported to result in quadriceps weakness, extensor lag, anterior knee pain, and lower functional scores, with the greatest impact after total patellectomy [5,27]. Where resection is unavoidable, partial patellectomy is preferred over total to preserve function [5]. When stable reconstruction is feasible, patella-preserving fixation is favoured, and comparative series report better extensor strength and function with fixation than with patellectomy in reconstructable comminuted patterns [27]. Contemporary reviews reflect this preference for preservation and careful patient selection [26]. Partial patellectomy involves resection of fracture fragments, with emphasis on preserving as much patella as possible and requires a concomitant advancement of the quadriceps or patella tendon to maintain continuation of the extensor mechanism [27]. A concise summary of surgical techniques, indications, and primary drawbacks is provided in Table 2 [26].

**Table 2:** Surgical management options for patellar fractures Abbreviations: ORIF=open reduction and internal fixation. Source: Baid et al. [26].

Technique	Implant / Notes	Indications	Primary drawback
ORIF - tension-band	Uses K-wires or cannulated screws with wire/suture in a figure-of-eight to create compression.	Indicated for transverse or simple fractures with adequate bone stock.	Symptomatic hardware irritation often requires later removal.
ORIF - cerclage wiring	Employs circumferential wire as an adjunct to other fixation for fragment containment.	Used as supplementary stabilisation in comminuted patterns.	Provides limited stability as a stand-alone construct and may irritate soft tissues.
ORIF - interfragmentary screws	Uses cannulated screws to deliver interfragmentary compression across the fracture line.	For simple transverse or vertical fractures that can be anatomically reduced.	May be less stable than tension-band constructs and risks fragment migration.
ORIF - locking plate fixation	Uses a fixed-angle plate allowing multiplanar screws and fragment-specific capture.	For comminuted fractures or osteoporotic bone requiring rigid fixation.	Can cause hardware prominence due to the subcutaneous location and sometimes needs removal.
Partial patellectomy	Resects a non-reconstructable pole and advances the patellar or quadriceps tendon to maintain continuity.	For inferior pole comminution not amenable to stable fixation.	Shortens the extensor mechanism and may lead to anterior knee pain or patella baja.
Total patellectomy	Excises the patella and advances or overlaps the tendons to restore continuity.	Reserved as salvage for non-reconstructable fractures or infection/tumour.	Leads to marked quadriceps weakness and poorer long-term function.

Figures 2-4 illustrate, respectively, a tension-band construct, cannulated screw fixation, and locking plate fixation [28].

Postoperative care and rehabilitation

Postoperative management of patellar fractures is similar in both non-operative and operative cases. For non-operative cases, patients are immobilised in extension with a splint or brace, followed by early physiotherapy to prevent stiffness, with radiographic follow-up to ensure stability [15]. Operative cases usually require a period of immobilisation initially depending on fracture patterns and fixation methods. Patients are usually placed in hinged knee braces, which may be locked in extension or allow limited flexion, with unrestricted weight-bearing permitted. Serial follow-up visits are arranged to gradually allow and increase the range of motion. Quadriceps strengthening may be initiated once pain improves and normal range of motion is established [15].

Outcomes and complications

Outcomes after patellar fracture depend on the fracture type and management approach. Simple, minimally displaced or vertical fractures with intact extensor mechanisms, managed non-operatively, generally achieve good to excellent outcomes in observational series [5,15]. After operative fixation, a meta-analysis of 15 studies reported a pooled non-union rate of 1.3% [19]. The main complication is hardware irritation due to the superficial position of the patella, often requiring removal once the fracture has healed, with re-operation rates exceeding 30% in some studies [15,19]. Across constructs, reoperation rates vary by implant and case mix. In direct comparative data, K-wire tension-band fixation has a higher implant-removal burden than screw-based tension bands, with elective removal reported as 37% after K-wire TBW versus 23% after cannulated-screw TBW in a 448-fracture series [29]. For locking plates, contemporary cohorts report lower return to theatre than classic K-wire constructs despite use in more comminuted patterns; one plating series of 85 fractures reported 9.41% overall reoperation and 2.35% specifically for painful hardware [30]. Taken together, hardware-related returns are typically higher with K-wire TBW, intermediate with screw-based tension bands, and lower with plate constructs, although differences in fracture pattern and follow-up must be considered. The incidence of re-operation has been shown to be lower in some studies where cannulated screws have been employed in the TBW construct instead of K-wires [31]. Furthermore, employing a combination of cannulated screws with suture (e.g. Fiberwire) rather than metallic wire can further reduce re-operation rates [32]. Other possible complications include post-traumatic arthritis of the patellofemoral joint leading to knee pain and decreased quality of life [33]. The incidence of arthritis varies and is highly dependent on degree of comminution of the initial injury, damage to articular cartilage, and quality of reduction of the fracture. Indeed, studies have shown a higher incidence of total knee arthroplasty being required in patients who have had patellar fractures in the past [34]. Although rare, patellectomy remains a possible management option. The main complication of partial or total patellectomy is quadriceps weakness, with reports of marked strength reduction and functional compromise [27]. Furthermore, as previously mentioned, the increased risk of requiring arthroplasty post patellar fracture has important considerations going forward, as this patient group will increase the burden of knee arthroplasties.

## Conclusions

Patellar fractures represent an important subset of knee injuries with significant implications for function and quality of life. Understanding the applied anatomy and biomechanics of the patella is essential for accurate diagnosis and effective management. While plain radiographs remain the mainstay of assessment, the increasing use of CT imaging has improved the detection of complex fracture patterns and informed treatment planning. The primary goal of management is to restore the extensor mechanism and preserve the patellofemoral joint. Non-operative care is effective for minimally displaced fractures with intact extensor function, whereas surgical intervention is indicated in displaced or unstable injuries. Although tension-band wiring has long been considered the gold standard, cannulated screws with suture augmentation and plate osteosynthesis are increasingly used, supported by biomechanical rationale and encouraging, though largely observational, clinical series suggesting fewer complications, while definitive superiority across indications has not been established. Direct randomised head-to-head comparisons are still lacking and no meta-analysis of randomised trials has been published to date. Priorities include pragmatic multicentre trials and robust registries with standardised functional outcomes, implant removal and re-operation endpoints, and sufficient long-term follow-up, with prespecified analyses in older and osteoporotic patients to establish optimal fixation methods accordingly.
